# Characterization of the CD49f^+^/CD44^+^/CD24^−^ single-cell derived stem cell population in basal-like DCIS cells

**DOI:** 10.18632/oncotarget.10203

**Published:** 2016-06-21

**Authors:** Nadire Duru, Ramkishore Gernapudi, Pang-Kuo Lo, Yuan Yao, Benjamin Wolfson, Yongshu Zhang, Qun Zhou

**Affiliations:** ^1^ Department of Biochemistry and Molecular Biology, Greenebaum Cancer Center, University of Maryland School of Medicine, Baltimore, MD 21201, USA

**Keywords:** tumor heterogeneity, single cell cloning, cancer stem cells, DCIS, non-coding RNAs

## Abstract

The molecular mechanisms responsible for the Ductal Carcinoma *in Situ* (DCIS)-Invasive Ductal Carcinoma (IDC) transition have yet to be elucidated. Due to the lack of molecularly targeted therapies, basal-like DCIS has a high risk of recurrence and progression to invasive and metastatic cancers. In this study, by applying a novel single-cell clonogenic approach with the CD49f^+^/CD44^+^/CD24^−^ surface markers, we characterized the aggressive clones that have enhanced self-renewal, migratory and invasive capacities derived from a human DCIS model cell line MCF10DCIS. The aggressive clones had elevated ALDH1 activity, lower global DNA methylation and increased expression of stem cell related genes, especially concurrent activation of SOX2/OCT4. In addition, we showed that the aggressive clones have increased expression of lincRNA-RoR and miR-10b compared to non-aggressive clones, which enhance their self-renewal and invasive abilities. Finally, we confirmed our *in vitro* results *in vivo,* demonstrating that aggressive clones were capable of forming tumors in nude mice, whereas non-aggressive clones were not. Our data suggest that lincRNA-RoR and miR10b could be used to distinguish aggressive clones from non-aggressive clones within the heterogeneous CD49f^+^/CD44^+^/CD24^−^ DCIS population. Our findings also provide the foundation to develop new chemoprevention agents for DCIS-IDC transition.

## INTRODUCTION

Ductal Carcinoma *In Situ* (DCIS) is an early non-invasive stage of breast cancer that is believed to be non-obligate precursor for invasive ductal carcinoma (IDC) [1]. The therapeutic standard of care for DCIS involves surgery, radiation, and in patients with hormone-responsive tumors, hormonal therapy [2]. Despite early detection and intervention, 15% of patients with DCIS show disease recurrence [3]. Very little is known regarding the molecular mechanisms responsible for DCIS progression. Currently, clinicians have no way of predicting which patients are at the greatest risk for disease recurrence or progression. Furthermore, while patients with basal-like DCIS pose the greatest risk for invasive progression, there are no available molecularly targeted therapies for basal-like DCIS [4].

Several recent studies have found that stem-like cells exist within heterogeneous DCIS lesions and may serve as malignant precursor cells for the transition to IDC. We recently isolated CD49f^+^/CD24^−^ stem-like cells from basal-like DCIS that possess high levels of ALDH1 activity and demonstrated self-renewal capacity *in vitro* and *in vivo* [4]. In addition, this stem-like subpopulation possessed enhanced migratory capacity compared to non-stem like cells, suggesting these cells might be disposed to malignant progression for IDC [5]. Finally, we found that this stem-like subpopulation could be targeted for differentiation with histone deacetylase (HDAC) inhibitors and DNA methyltransferase (DNMT) inhibitors, resulting in activation of tumor suppressor miR-140 [5].

Since different tumor cell populations have different potentials for tumor initiation, metastasis, angiogenesis and therapeutic resistance, one of the largest challenges in designing the treatment plan for cancer patients is tumor heterogeneity. Several studies demonstrated that cancer stem cells (CSCs) and clonal evolution contribute tumor heterogeneity [6]. Technological advances have made high-throughput tumor genome sequencing possible, leading to the merging of ideas on hierarchical cancer stem cell and tumor clones. Tumor heterogeneity is likely due to the combination of genetic, epigenetic and micro-environmental stimuli acting on CSCs, leading to the development of multiple clones with functional variations within CSC subpopulations [7]. Currently, most cancer research is conducted with whole-population based cell models; hence the data obtained do not address the behavior of individual clones. Unlike whole-population based research, single-cell approaches will eliminate the issues of heterogeneity and cellular hierarchy within the tumor, enabling researchers to study and target specific cancer cell populations of interest. Clonal analysis using a single cell approach was recently performed in glioblastoma (GBM) tumor samples where single cell derived clones were compared to each other for their phenotypic and genomic properties to identify the tumorigenic and drug-resistant clones [8]. Another recent study performed single-cell gene-expression experiments via PCR array, and revealed that early stage metastatic cells display a distinct gene expression profile especially for the expression of the genes associated with stem cells, epithelial-to-mesenchymal transition (EMT), pro-survival and dormancy [9].

MCF10DCIS is a model cell line of poorly differentiated basal-like ductal carcinoma *in situ* that forms DCIS lesions when injected into the mammary gland of nude mice [10]. In the present study, we used a single-cell approach to select the most aggressive clones from the CD49f^+^/CD44^+^/CD24^−^ MCF10DCIS stem cell population for *in vitro* and *in vivo* characterization. We found that the aggressive clones derived from the CD49f^+^/CD44^+^/CD24^−^ DCIS stem cell population had higher ALDH1 activity, lower global DNA methylation and expressed significantly higher levels of stem cell related proteins such as SOX2, OCT4 and SOX9. We identified lincRNA-RoR and miR-10b as key molecules to increase self-renewal, migratory, and invasive capacities of aggressive clones. Finally, our *in vivo* studies confirmed that the aggressive clones had higher tumorigenic capacity.

## RESULTS

### CD49f^+^/CD24^−^ single-cell derived clones have different self-renewal and invasion capabilities

**Figure 1:**
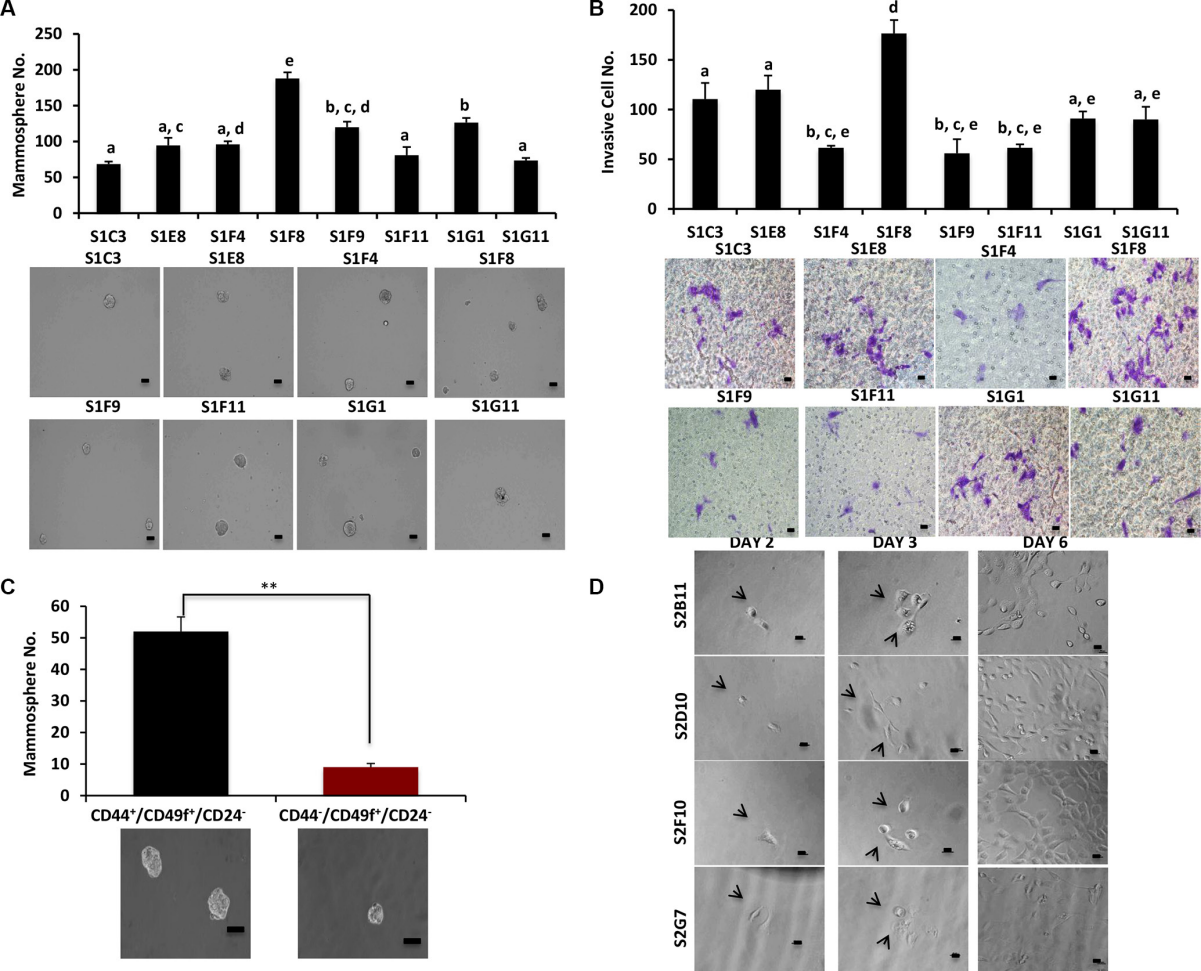
CD49f^+^/CD24^−^ single-cell derived clones have different self-renewal and invasion capabilities (**A**), Mammosphere formation was evaluated in different clones that were derived from CD49f^+^/CD24^−^ single cells. Clones showed a big variation in terms of their mammosphere forming capacity. Bar scale represents 50 μm. One-way ANOVA is used to perform the correlation analysis. Samples with no statistically significant differences are placed in the same letter group. Differentiated groups have at least a *p* value of less than 0.05. (F stat = 53.45; *p* value = 4.3e-06; df = 7). (**B**) The ability of the CD49f^+^/CD24^−^ single cell derived clones to invade was assessed via transwell invasion assay. The clones showed a variation in terms of their invading capacity and the invasion pattern of the clones matched their mammosphere formation ability. Bar scale represents 25 μm. One-way ANOVA is used to perform the correlation analysis. Samples with no statistically significant differences are placed in the same letter group. Differentiated groups have at least a *p* value of less than 0.05. (F stat = 24.07; *p* value = 9.04e-05; df = 7). (**C**) MCF10DCIS parental cells were sorted into two groups CD49f^+^/CD44^+^/CD24^−^ and CD49f^+^/CD44^+^/CD24^−^ and used for mammosphere formation assay to understand the role of CD44 in self-renewal of DCIS cells. Bar scale represents 50 μm. Data represents the mean ±S.D (*n* = 3); ^**^p < 0.01. (**D**) Representative images of single-cell sorting process from 4 clones that were used extensively in the future experiments. The images show the cells at day 1 as single cells or single cells getting ready to divide as well as the progress of the single cells to form the clones in different days. Bar scale represents 25 μm.

We previously identified a stem-like cell subpopulation of MCF10DCIS with CD49f^+^/CD24^−^ phenotype that possess high levels of ALDH1 activity and have self-renewal capacity *in vitro* and *in vivo* [4]. We hypothesized that this specific subpopulation drives the tumorigenesis and progression of DCIS. To further characterize this heterogeneous stem cell population, we performed a Fluorescent-Activated Cell Sorting (FACS) based single-cell approach to sort CD49f^+^/CD24^−^ single cells from MCF10DCIS cells into 96-well plates, and generated 25 CD49f^+^/CD24^−^ single-cell derived clones. We next characterized the individual clones for traits associated with aggressiveness. When grown in attachment-free mammosphere conditions, the clones displayed a high variation in mammosphere formation (Figure 1A). The clone with the highest self-renewal, S1F8, was also the most invasive as determined by a transwell invasion assay (Figure 1B). These data demonstrate that the heterogeneity of the CD49f^+^/CD24^−^ stem-like cell subpopulation (Table 1) contributes to the formation of individual clones with different stem cell self-renewal and cell invasion abilities.

**Table 1:** CD49f^+^/CD24^−^ and CD49f^+^/CD44f^+^/CD24^−^ single cell derived clones and their phenotypes

Markers	Clones	Average Mammosphere No.	Average Migrated Cell No.	Average Invasive Cell No.	Average Global Methyl.	Phenotype
CD49f^+^/CD24^−^	S1C3	69	N/A	110	N/A	Less aggressive
	S1E8	95	N/A	120	N/A	More aggressive
	S1F4	96	N/A	61	N/A	Less aggressive
	S1F8	188	1230	176	0.82	More aggressive
	S1F9	120	815	56	0.36	Less aggressive
	S1F11	81	N/A	61	N/A	Less aggressive
	S1G1	127	N/A	91	N/A	Lessaggressive
	S1G11	74	N/A	90	N/A	Less aggressive
CD49f+/CD44^+^/CD24^−^	S2B11	475	2400	123	0.39	More aggressive
	S2D10	120	1067	67	0.70	Less aggressive
	S2D11	192	1730	134	0.32	More aggressive
	S2F5	151	1103	260	0.56	Less aggressive
	S2F10	80	290	43	0.71	Less aggressive
	S2G7	172	1375	145	0.24	More aggressive
	S2H4	189	N/A	101	N/A	More aggressive

CD49f in combination with CD44^high^/CD24^low^ has been used as predominant stem cell markers for separation of aggressive breast tumor cells [11]. We decided to incorporate CD44 marker for further characterization of our single-cell derived clones. We observed that CD49f^+^/CD44^+^/CD24^−^ cells formed significantly higher numbers of mammospheres (5-fold) compared to CD49f^+^/CD44^−^/CD24^−^/ cells (Figure 1C), supporting that CD44 is a critical marker for studies of single cell clone behaviors. We sorted MCF10DCIS cells for single cells with CD49f^+^/CD44^+^/CD24^−^, and generated 21 clones. These individual clones showed varied clonogenic and proliferative abilities (Table 1). Figure 1D shows a representative image of the clonogenic expansion from single cells of 2 more aggressive (S2B11 and S2G7) and 2 less aggressive clones (S2D10 and S2F10) that were used extensively in further experiments. Collectively these data confirm the heterogeneity of breast cancer stem cell population.

### Different mammosphere formation ability and DNA methylation in CD49f^+^/CD44^+^/CD24^−^ single-cell derived clones

**Figure 2:**
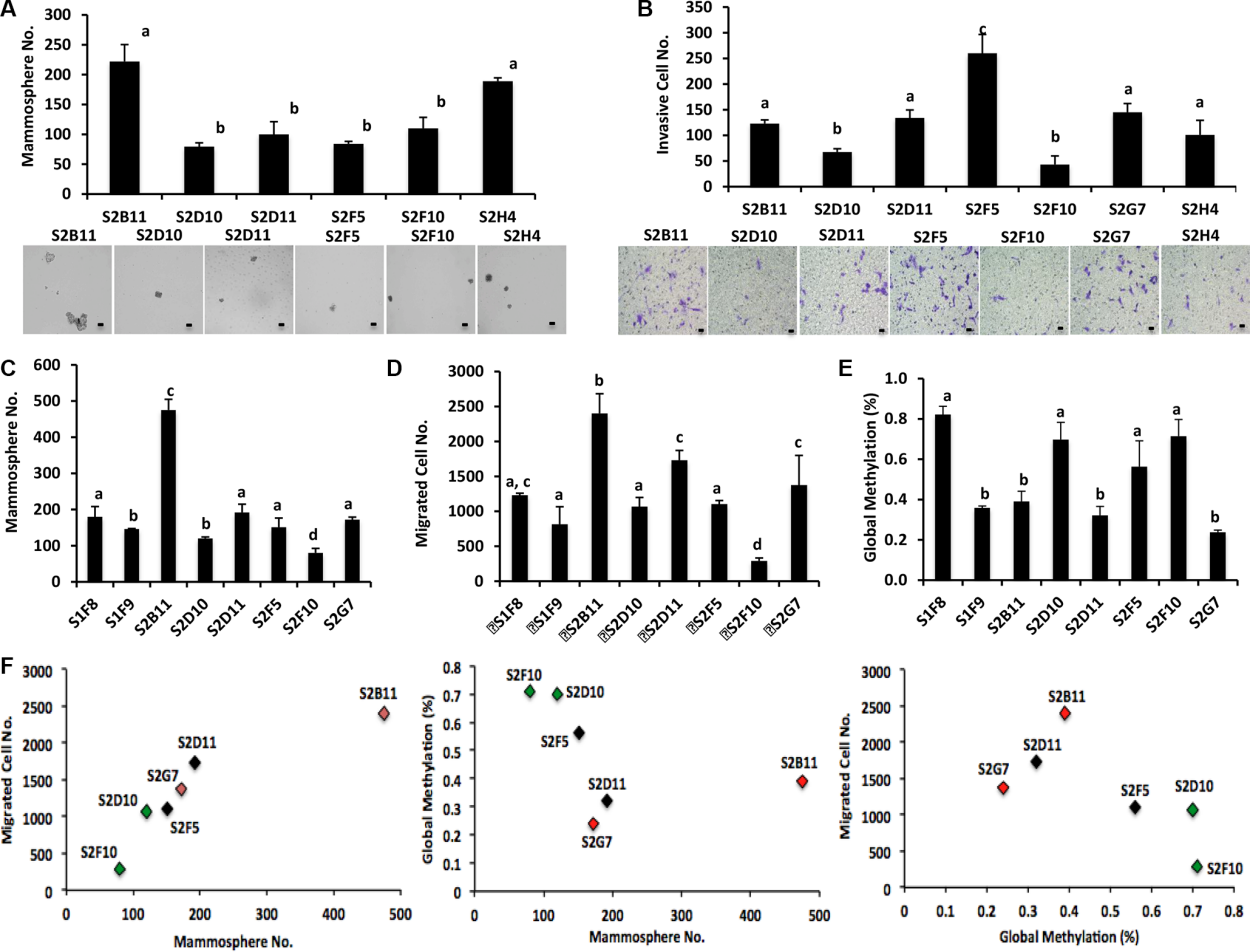
Different mammosphere formation ability and DNA methylation in CD49f^+^/CD44^+^/CD24^−^ single-cell derived clones (**A**) Mammosphere formation was evaluated in different clones that were derived from CD49f^+^/CD44^+^/CD24^−^ single cells. Clones showed a big variation in terms of their mammosphere forming capacity. Bar scale represents 50 μm. One-way ANOVA is used to perform the correlation analysis. Samples with no statistically significant differences are placed in the same letter group. Differentiated groups have at least a *p* value of less than 0.05. (F stat = 25.61; *p* value = 0.0006; df = 5). (**B**) The ability of the CD49f^+^/CD44^+^/CD24^−^ single cell derived clones to invade was assessed via transwell invasion assay. The clones showed a variation in terms of their invading capacity and the invasion pattern of the clones matched their mammosphere formation ability. Bar scale represents 25 μm. One-way ANOVA is used to perform the correlation analysis. Samples with no statistically significant differences are placed in the same letter group. Differentiated groups have at least a *p* value of less than 0.05. (F stat = 67.05; *p* value = 1.3e-12; df = 6). (**C**) Mammosphere formation ability of CD49f^+^/CD44^+^/CD24^−^ single cell derived clones was compared to the mammosphere formation ability of CD49f^+^/CD24^−^ single cell derived clones. CD49f^+^/CD44^+^/CD24^−^ single cell derived clones formed more and bigger mammospheres compared to most aggressive CD49f^+^/CD24^−^ single cell derived clones. One-way ANOVA is used to perform the correlation analysis. Samples with no statistically significant differences are placed in the same letter group. Differentiated groups have at least a *p* value of less than 0.05. (F stat = 75.92; *p* value = 1.1e-06; df = 7) (**D**) The migration capacity of CD49f^+^/CD44^+^/CD24^−^ single cell derived clones was compared to the migration capacity of CD49f^+^/CD24^−^ single cell derived clones. CD49f^+^/CD44^+^/CD24^−^ single cell derived clones migrated more compared to most aggressive CD49f^+^/CD24^−^ single cell derived clones. One-way ANOVA is used to perform the correlation analysis. Samples with no statistically significant differences are placed in the same letter group. Differentiated groups have at least a *p* value of less than 0.05. (F stat = 34.43; *p* value = 5.1e-11; df = 7). (**E**) Global DNA methylation status of CD49f^+^/CD44^+^/CD24^−^ single cell derived clones was compared to the global methylation status of CD49f^+^/CD24^−^ single cell derived clones. The clones showed a variation in terms of their global DNA methylation status but in general more aggressive clones had a lower global DNA methylation compared to less aggressive clones. One-way ANOVA is used to perform the correlation analysis. Samples with no statistically significant differences are placed in the same letter group. Differentiated groups have at least a *p* value of less than 0.05. (F stat =12.35; *p* value = 1.3e-06; df = 7). (**F**) Corelation analyses show a strong correlation between mammosphere formation ability and migration capacity of CD49f^+^/CD44^+^/CD24^−^ single cell derived clones (R^2^ = 0.9, *p* < 0.01). The correlation coefficient is still on the positive side but not significant for the relationship between global methylation of these clones and their mammosphere formation and migration capacity (R^2^ = 0.4 and R^2^ = 0.5, respectively).

We used CD49f^+^/CD44^+^/CD24^−^ single cell derived clones and examined stem cell self-renewal using a mammosphere assay. The clone S2B11 formed significantly more and bigger mammospheres compared to the clone S2D10 (Figure 2A). We then performed the *in vitro* invasion assay and found that S2B11 had a higher invasive capacity compared to S2D10 (Figure 2B). In addition, the CD49f^+^/CD44^+^/CD24^−^ single cell derived clone S2B11 had a significantly enhanced mammosphere formation and cell migration ability compared to S1F8, the most aggressive clone derived from the CD49f^+^/CD24^−^ stem cell population (Figures 2C and 2D), suggesting that the clone S2B11 derived from CD49f^+^/CD44^+^/CD24^−^ single cell represent the aggressive phenotype of cancer stem cells within the CD44^+^/CD24^−^ subpopulation. It is well known that cancer cells undergo epigenetic changes, which might be one of the key events for initiation and progression of cancer [12]. To investigate the epigenetic changes in the aggressive and non-aggressive clones, we examined the global DNA methylation status of these clones. We found that the aggressive clones have significantly lower DNA methylation compared to non-aggressive clones (Figure 2E). The correlation analyses showed a strong relationship between migration capacity and mammosphere formation ability of CD49f^+^/CD44^+^/CD24^−^ single cell derived clones. The correlation between global methylation of these clones and their migration and mammosphere formation capacity was also positive (Figure 2F). These data demonstrate that DNA hypo-methylation may contribute to the rapid cell growth and both self-renewal and migration capacities are significantly enhanced in the aggressive clones.

### CD49f^+^/CD44^+^/CD24^−^ single-cell derived clones retain their characteristics at further passages

**Figure 3:**
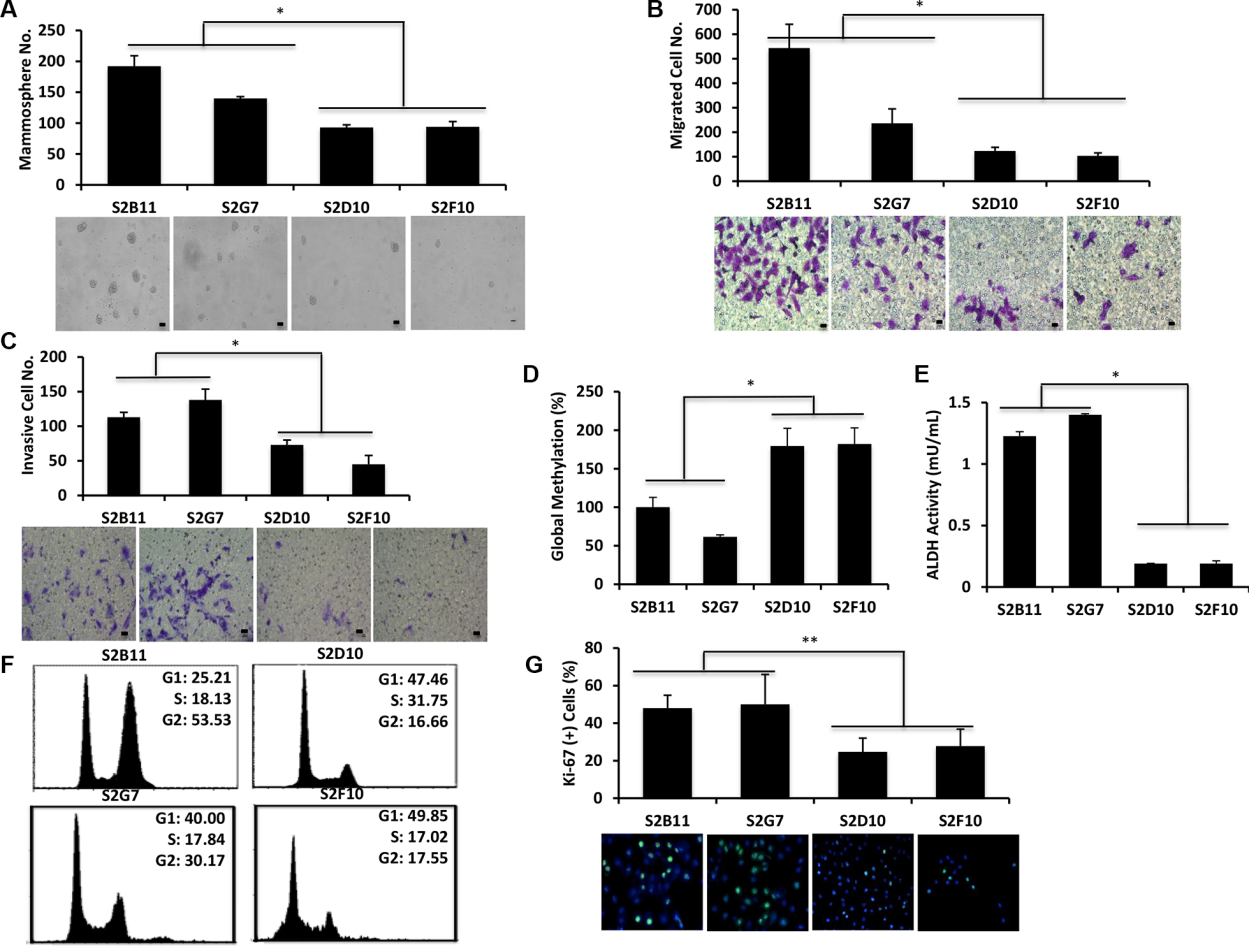
CD49f^+^/CD44^+^/CD24^−^ single-cell derived clones retain their characteristics at further passages Functional assays were performed using 2 most aggressive (S2B11 and S2G7) and 2 less aggressive (S2D10 and S2F10) clones that were derived from CD49f^+^/CD44^+^/CD24^−^ single cells after passage 10. (**A**) More aggressive clones still formed significantly more and bigger spheres compared to less aggressive clones. Bar scale represents 50 μm. (**B**) More aggressive clones had significantly higher number of migrated cells compared to less aggressive clones. Bar scale represents 25 μm. (**C**) More aggressive clones had significantly higher number of invasive cells compared to less aggressive clones. Bar scale represents 25 μm. (**D**) More aggressive clones had significantly lower global DNA methylation compared to less aggressive clones. (**E**) More aggressive clones had significantly higher ALDH activity than less aggressive clones. (**F**) More aggressive (S2B11 and S2G7) and less aggressive (S2D10 and S2F10) clones showed a different pattern of cell cycle. S2B11 and S2G7 had a significantly higher number of cells accumulated in G2 phase compared to S2D10 and S2F10. G, S2B11 and S2G7 cells are more prolific indicated by their higher staining of Ki-67 compared to S2D10 and S2F10 cells. Data represents the mean ± S.D (*n* = 3); ^*^p < 0.05.

Next, we examined the properties of our single-cell clones at different passage numbers to ensure the observed functional effects are maintained. We chose two of the most aggressive (S2B11 and S2G7) and two of the non-aggressive (S2D10 and S2F10) CD49f^+^/CD44^+^/CD24^−^ clones for long-term culture. At passage 10, we repeated the functional assays performed in Figure 2 to determine whether the clones maintained their differential phenotypes. The aggressive clones formed significantly more and bigger mammospheres compared to non-aggressive ones (Figure 3A). Additionally, they migrated (Figure 3B) and invaded (Figure 3C) at a higher rate than the non-aggressive clones and had significantly lower global methylation compared to non-aggressive clones (Figure 3D). These results confirm that the single-cell derived clones retain their properties *in vitro*. ALDH1 is a detoxifying enzyme that is related to drug resistance, and is a well-established marker for breast cancer stem cells [13]. Using an ALDH1 activity assay, we found that aggressive clones had a 6-fold increase in ALDH1 activity compared to non-aggressive clones (Figure 3E). As deregulated proliferation is one of the primary hallmarks of cancer, we compared the cell cycle status of the most aggressive clones (S2B11 and S2G7) to the least aggressive clones (S2D10 and S2F10). The non-aggressive S2D10 and S2F10 clones had a more stereotypical cell cycle pattern, with the majority of cells in the G1 phase and minority in the G2 (16.66% and 17.55%, respectively). Interestingly, in the aggressive S2B11 clone, the majority of the cells were in the G2 phase (53.53%). In the S2G7 clone the cells that were in G2 phase (30.17%) were almost double of the cells that were in G2 phase for the non-aggressive clones (Figure 3F). We hypothesized that the accumulation in G2 was either due to apoptotic resistance, or higher proliferation. With an Annexin V assay we found there was no change in the rate of apoptosis between aggressive and non-aggressive clones (data not shown). We next examined proliferation using immunofluorescent staining for Ki-67. As seen in Figure 3G, Ki-67 staining in aggressive S2B11 and S2G7 clones were significantly higher compared to non-aggressive S2D10 and S2F10 clones, indicating that the S2B11 and S2G7 populations undergo rapid cell proliferation, which would be advantageous for tumor initiation and progression.

### Overexpression of K14/ARF6 in aggressive CD49f^+^/CD44^+^/CD24^−^ single-cell derived clones

**Figure 4:**
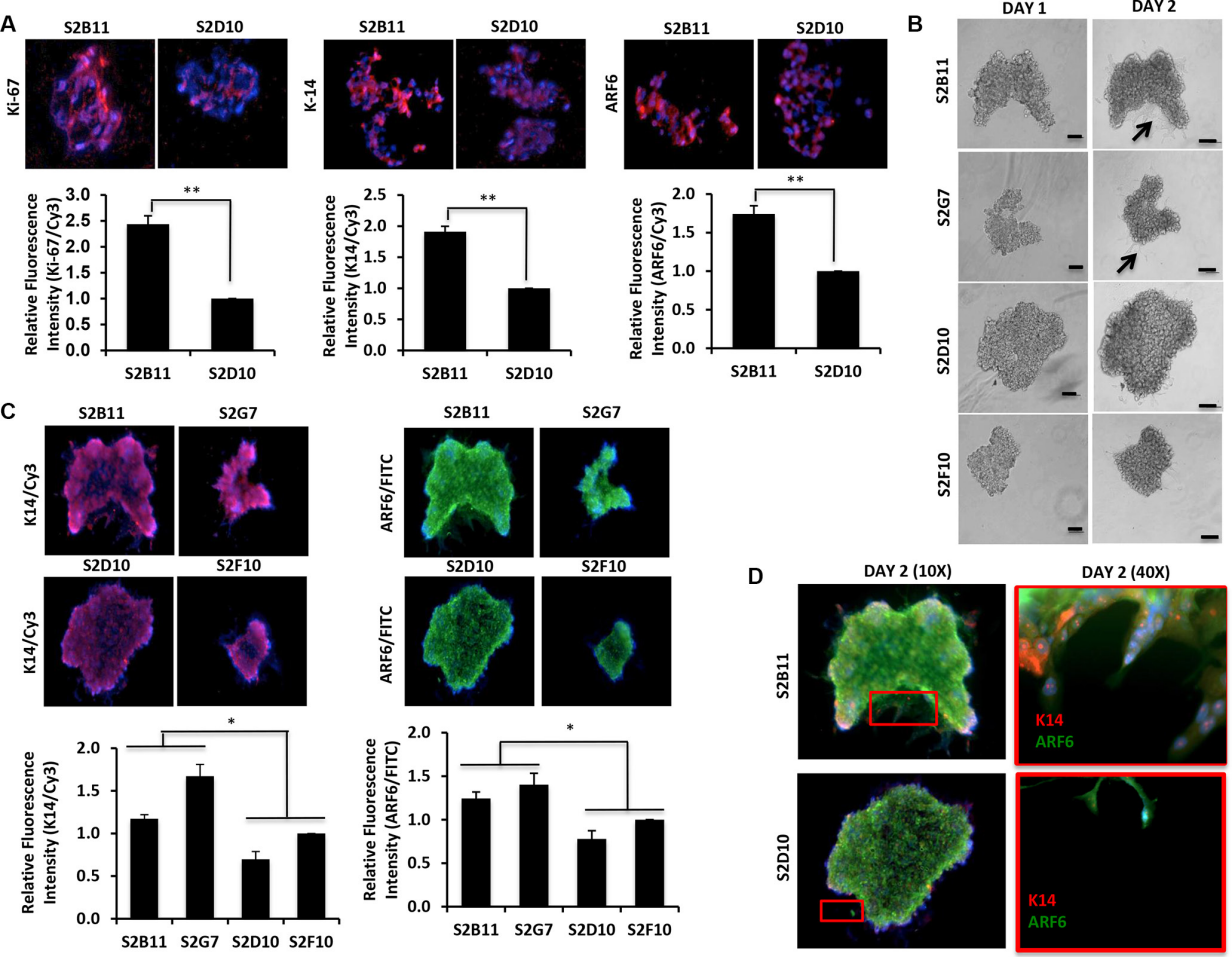
Overexpression of K14/ARF6 in aggressive CD49f^+^/CD44^+^/CD24^−^ single-cell derived clones (**A**) Sections of S2B11 aggressive clone spheres have higher expression of Ki-67, K14 and ARF6 compared to spheres of non-aggressive S2D10 clone. (**B**) Spheres of more aggressive (S2B11 and S2G7) and less aggressive (S2D10 and S2F10) clones were embedded into 3D matrigel and their invasiveness was monitored. Spheres of more aggressive clones formed protruding structures showing their invading capacity while the less aggressive clones failed to form such structures. Bar scale represents 100 μm. (**C**) Spheres were stained with antibodies against K14 (red) and ARF6 (green). S2B11 and S2G7 have elevated K14 and ARF6 expression levels compared to S2D10 and S2F10. (**D**) Zoomed in image of S2B11 shows that the leading cells are either K14 or ARF6 positive if they are not positive for both. S2D10 failed to form leading cells.

Recently, the specific invasive leading cells were observed in most breast cancer subtypes. These cells are present on the leading edge of invasive spheroids, and are identified by high expression of K14, a basal-lineage associated cytokeratin [14]. ADP-ribosylation factor 6 (ARF6), another critical protein in invasive breast cancer cells [15], enhances tumor invasion through the regulation of E-cadherin localization and cell-cell adhesion in triple-negative breast cancer [16]. Therefore, we decided to test if K14 and ARF6 are associated with aggressive clones. We performed immunofluorescence staining for Ki-67, K14 and ARF6 on clone spheroids grown in matrigel 3D-culture. The expressions of all three markers were increased significantly in S2B11 spheres compared to S2D10 spheres (Figure 4A). Next, we tested the differentiation capacity of the clones using a 3D matrigel cell culture system to determine the role of our aggressive clones in invasion. We found that aggressive clones (S2B11 and S2G7) formed more protrusive structures compared to non-aggressive clones (S2D10 and S2F10) in the 3D matrigel environment (Figure 4B). We then stained the spheres in matrigel with K14 and ARF6. We found that spheres formed by aggressive clones had significantly higher expression of K14 and ARF6 compared to the spheres formed by non-aggressive clones (Figure 4C). Moreover, we observed that only aggressive clones show the leading cells that are positive for both K14 and ARF6, suggesting that K14 and ARF6 may contribute to the invasive abilities of aggressive clones (Figure 4D).

### The SOX2/OCT4 signaling axis is upregulated in aggressive CD49f^+^/CD44^+^/CD24^−^ single-cell derived clones

**Figure 5:**
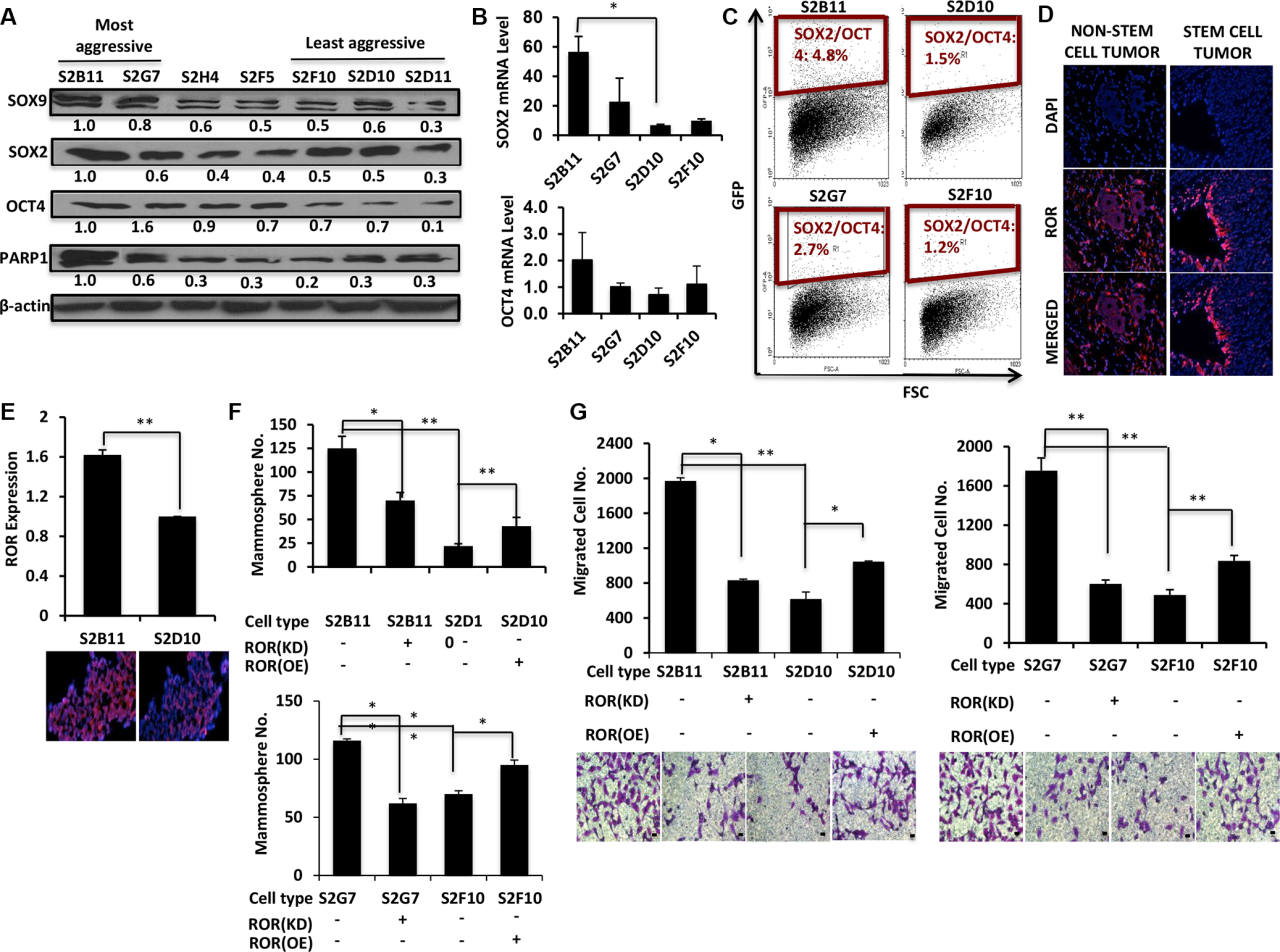
SOX2/OCT4/lincRNA-RoR signaling axis is upregulated in aggressive CD49f^+^/CD44^+^/CD24^−^ single-cell derived clones (**A**) CD49f^+^/CD44^+^/CD24^−^ single cell derived clones were categorized as more aggressive (S2B11 and S2G7), medium aggressive (S2H4 and S2F5) and less aggressive (S2D10, S2F10 and S2D11) based on *in vitro* functional studies and tested for the protein expression of stem cell related genes SOX9, SOX2, OCT4 and PARP1. More aggressive clones had a higher expression of these proteins. (**B**) mRNA expression levels of SOX2 and OCT4 was assessed with qRT-PCR in S2B11, S2G7, S2D10 and S2F10. The expression level was higher in S2B11 and S2G7 compared to S2D10 and S2F10. (**C**) A dual reporter plasmid for SOX2/OCT4 expression was used to check weather the activation of both these genes was a factor in the aggressiveness of the clones. (**D**) Comparison of RoR expression in tumors of parental non-sorted MCF10DCIS cells to the tumors of stem cell-like MCF10ADCIS cells (CD49f^+^/CD24^−^). Expression of RoR is increased and organized along the ducts in tumor sections of stem cell-like MCF10DCIS cells. (**E**) qRT-PCR shows the elevated ROR expression level in more aggressive clone S2B11 compared to less aggressive clone S2D10. Data represents the mean ± S.D (*n* = 2). Data is supported with fluorescence *in situ* hybridization of lincRNA-RoR in S2B11 and S2D10 clones. (**F**–**G**) Mammosphere formation ability (Bar scale represents 50 μm) (F) and migration capacity (Bar scale represents 25 μm) (G) was evaluated in S2B11, S2G7, S2D10 and S2F10 after knocking-down the ROR in S2B11 and S2G7 clones with shRoR and overexpressing the RoR in S2D10 and S2F10 clones. Data represents the mean ± S.D (*n* = 3); ^*^p < 0.05, ^**^p < 0.01.

Next, we wanted to examine the specific genes related to stemness and aggressiveness in breast cancer that might have been activated in these clones. We previously demonstrated that DCIS stem-like cells have enhanced activation transcription factors SOX9 and SOX2 [5], both of which increase the self-renewal and mammosphere formation. Therefore, we first compared the protein expression levels of SOX9 and SOX2 in CD49f^+^/CD44^+^/CD24^−^ single cell derived clones. We found that aggressive clones expressed higher levels of SOX9 and SOX2 compared to non-aggressive clones. (Figure 5A). We next assessed the protein expression level of OCT4, a transcription factor that interacts with SOX2 and is necessary to maintain stem cell pluripotency [17]. As expected the expression of OCT4 was also higher in aggressive clones compared to non-aggressive clones (Figure 5A). In addition, mRNA levels of SOX2 and OCT4 were higher in aggressive clones (Figure 5B). Finally, we examined the expression levels of PARP1, which is associated with proliferation, apoptosis, transcriptional regulation, DNA repair, and malignant transformation. We found that PARP1 is overexpressed in aggressive clones compared to non-aggressive clones (Figure 5A). We then hypothesized that concurrent activation of both SOX2 and OCT4 may increase the aggressiveness of CD49f^+^/CD44^+^/CD24^−^ single-cell derived clones. We used a novel SOX2/OCT4 GFP reporter plasmid that fluoresces only when both SOX2 and OCT4 are expressed [18] to transfect S2B11, S2G7, S2D10 and S2F10 cells and performed FACS to quantify the GFP-positive subpopulations. The aggressive clones showed an increase in SOX2^+^/OCT4^+^ cells compared to the non-aggressive clones (Figure 5C). These results suggest that aggressive clones activate SOX2/OCT4 signaling, which maintain stem cell self-renewal.

### LincRNA-RoR increases the self-renewal and migration of aggressive CD49f^+^/CD44^+^/CD24^−^ single-cell derived clones

The long-intergenic noncoding RNA Regulator of Reprogramming (lincRNA-RoR) has been shown to be essential for stem cell pluripotency [19]. Additionally, lincRNA-RoR is a competitive-endogenous lincRNA that shares miRNA response elements with key stemness and invasive proteins including OCT4, SOX2 and ARF6 and prevents miRNA-mediated suppression of these key transcription factors [20–21]. Moreover, overexpression of lincRNA-RoR leads to increased self-renewal in both normal and neoplastic mammary stem cells [22]. Given that SOX2 and OCT4 are overexpressed in the aggressive clones we investigated the role of RoR in the aggressiveness and stemness of our single-cell clones. First, we used immunofluorescence to compare RoR expression in tumors formed from parental non-sorted MCF10DCIS cells to tumors formed from CD49f^+^/CD24^−^ stem-like cells. RoR expression was dramatically higher in the CD49f^+^/CD24^−^ tumor and it was mainly expressed along the ducts (Figure 5D). We next used RT-PCR to compare the expression profile of RoR in the single-cell derived aggressive and non-aggressive clones. We found that RoR was significantly upregulated in the more-aggressive S2B11 clonal population compared to non-aggressive S2D10, and confirmed this result using fluorescence *in situ* hybridization of lincRNA-RoR in S2B11 and S2D10 clones (Figure 5E). To test whether RoR is responsible for the increased mammosphere formation of aggressive clones (Figure 3A), we used shRoR to knock down RoR in S2B11 and S2G7 cells. Mammosphere formation was significantly decreased. In contrast, mammosphere formation was increased significantly in the non-aggressive clones S2D10 and S2F10 with overexpressed RoR (Figure 5F). Using these same clones, we next measured their migratory abilities. Knockdown of RoR in the S2B11 and S2G7 clones significantly decreased the number of migrated cells. The S2D10 and S2F10 clones with overexpressed RoR caused a significant increase in the number of migrated cells (Figure 5G). These results show that RoR increases the self-renewal and migration in the aggressive clones derived from DCIS stem cells.

### miR-10b increases migration and invasion capacity of aggressive CD49f^+^/CD44^+^/CD24^−^ single-cell derived clones

**Figure 6:**
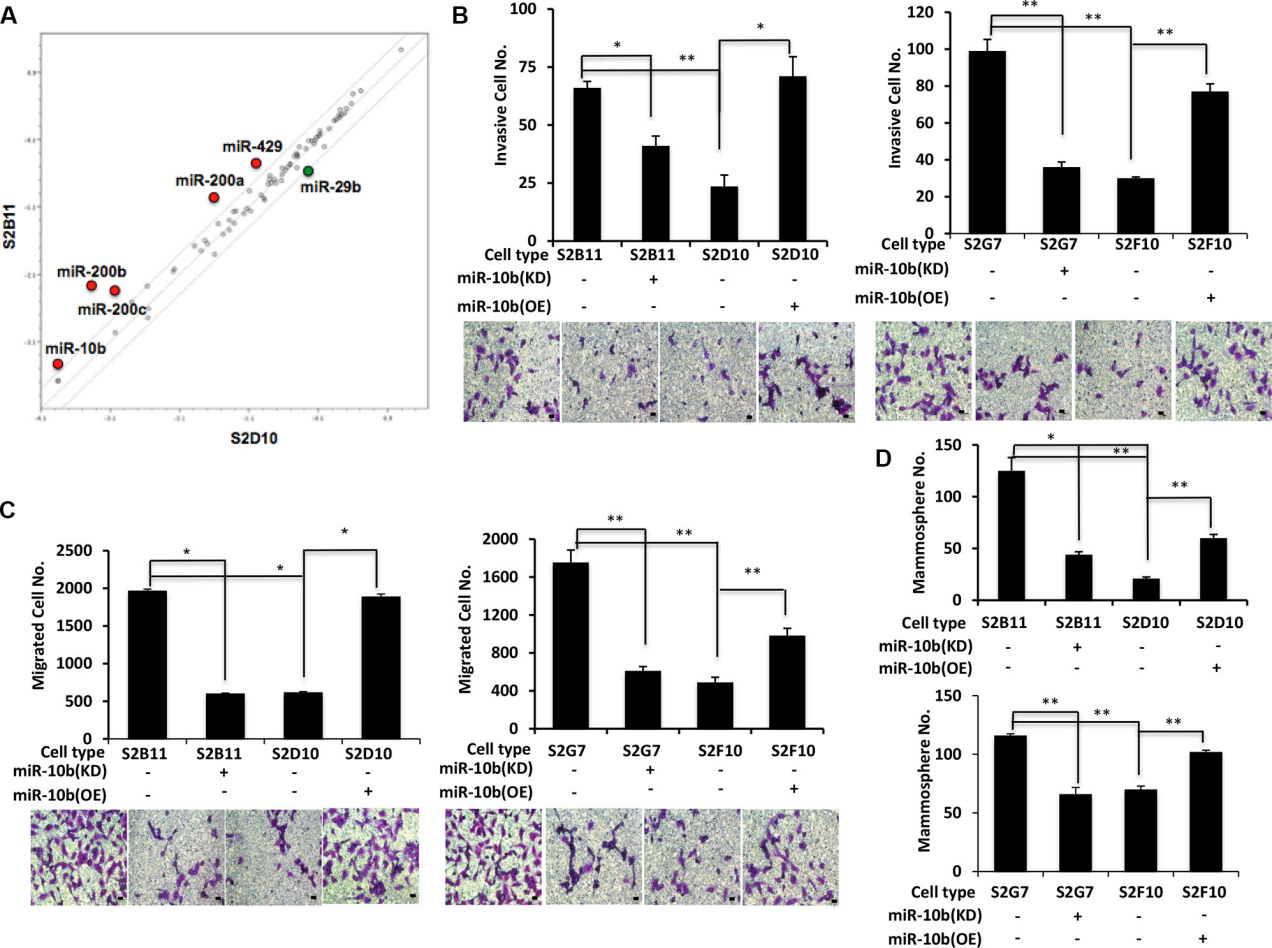
miR-10b is a key regulator for of migration and invasion capacity in aggressive CD49f^+^/CD44^+^/CD24^−^ single-cell derived clones (**A**) Comparative human breast cancer-associated microRNA expression levels in S2B11 and S2D10 clones. (**B**–**D**) Invasion capacity (B), migration capacity (Bar scale represents 25 μm) (C) and mammosphere formation ability (Bar scale represents 50 μm) (D) of S2B11, S2G7, S2D10 and S2F10 was evaluated after knocking-down the miR-10b in S2B11 and S2G7 clones with sponge miR-10b and overexpressing the miR-10b in S2D10 and S2F10 clones. Data represents the mean ± S.D (*n* = 2); ^*^p < 0.05, ^**^p < 0.01.

To elucidate the pathways conferring migration and invasion to aggressive CD49f^+^/CD44^+^/CD24^−^ single-cell derived clones, we used a PCR array to screen miRNAs in aggressive clone that are associated with breast cancer S2B11 and non-aggressive clone S2D10 and identified six miRNAs that were differentially expressed between these clones. Five of these miRNAs were upregulated (miR-429, miR-200a, miR-200b, miR-200c and miR-10b) and one of them was downregulated (miR29-b) in the aggressive S2B11 clone compared to S2D10 (Figure 6A). MiR-10b is upregulated in DCIS lesions compared to normal breast tissue [23], and miR-10b overexpression is associated with enhanced cell migration and invasion in breast cancer [24]. Based on these previous reports and given that our aggressive S2B11 clone had increased miR-10b expression, we hypothesized that miR-10b might have a key role in maintenance of the aggressive behavior of our clones. We first tested whether miR-10b is responsible for the increased invasive capacity of the aggressive clones (Figure 3C). When miR-10b was knocked down in the S2B11 and S2G7 clones, their invasive ability was significantly decreased, whereas upon miR-10b overexpression the invasive capacity of S2D10 and S2F10 clones increased significantly (Figure 6B). We next used the same cells to measure their migration levels. Knockdown of miR-10b in S2B11 and S2G7 clones decreased significantly the number of migrated cells and overexpressing miR-10b in S2D10 and S2F10 clones caused a significant increase in the number of migrated cells (Figure 6C). Finally, we wanted to see if miR-10b has also a role in the self-renewal of our aggressive clones. When miR-10b was knocked down in the S2B11 and S2G7 clones, mammosphere formation was significantly decreased, whereas upon miR10b overexpression mammosphere formation increased significantly in S2D10 and S2F10 clones (Figure 6D). These results demonstrate that miR-10b enhances the migration and invasion capacity of single-cell derived clones.

### Aggressive CD49f^+^/CD44^+^/CD24^−^ single-cell derived clones show tumorigenesis *in vivo*

**Figure 7:**
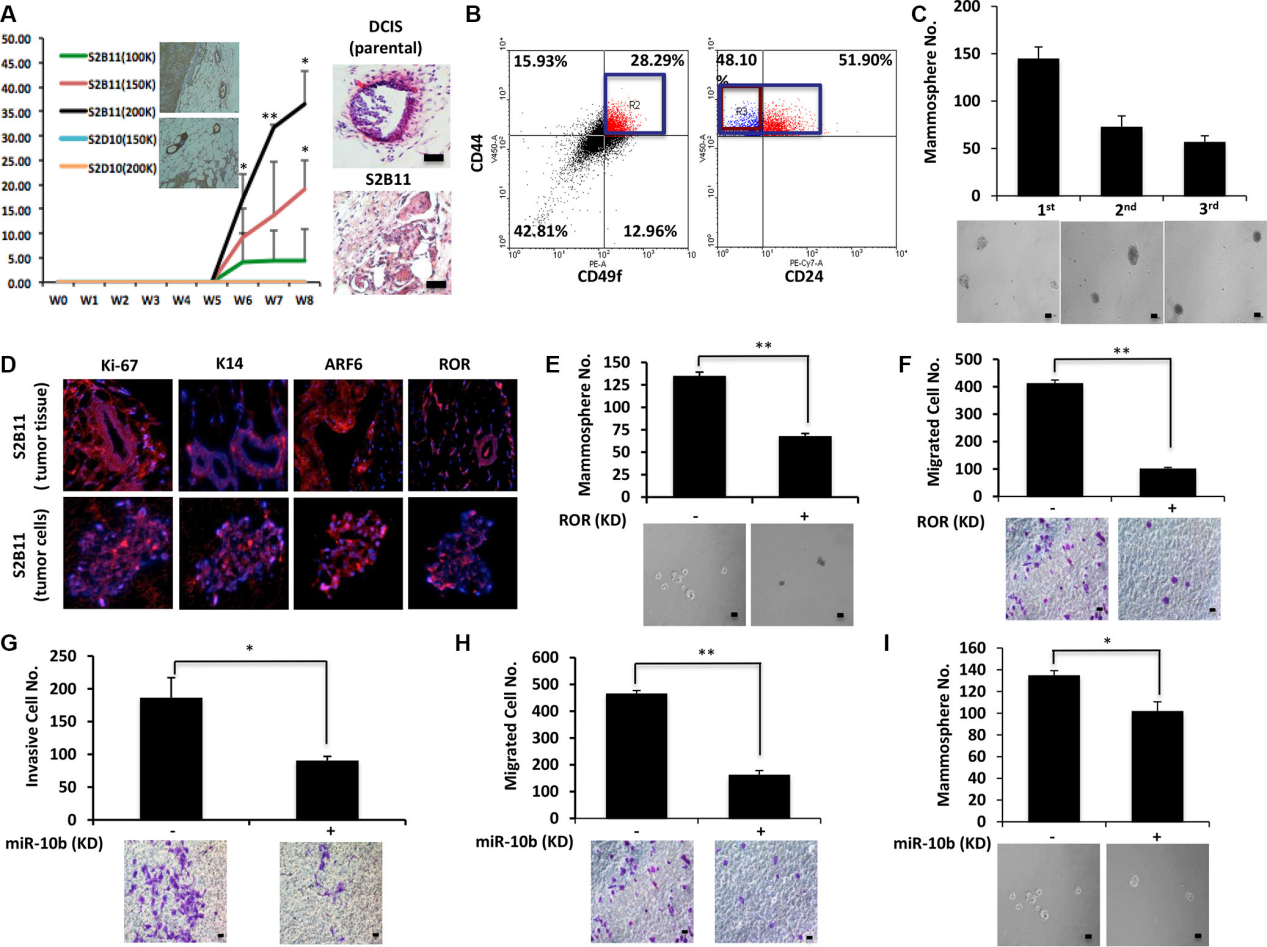
Aggressive CD49f^+^/CD44^+^/CD24^−^ single-cell derived clones show tumorigenesis *in vivo* (**A**) Athymc nude mice were injected with 2 × 10^5^, 1.5 × 10^5^ and 1 × 10^5^ of S2B11 cells and 2 × 10^5^ and 1.5 × 10^5^ of S2D10 cells. All mice that were injected with S2B11 cells formed tumors and none of the mice that were injected with S2D10 cells showed any tumor formation. The high expression level of K14 was confirmed in S2B11 tumors via IHC. H&E staining showed the more invasive nature of the S2B11 tumors. Bar scale represents 100 μm. Data represents the mean ±S.D (*n* = 10); ^*^p < 0.05, ^**^p < 0.01. (**B**) CD49f^+^/CD44^+^/CD24^−^ stem cell-like subpopulation in S2B11 tumors was confirmed via FACS analysis showing that this subpopulation was consisting 13.6% of whole tumor cell population. (**C**) Mammosphere formation ability of the primary S2B11 tumor cells was evaluated for 3 generations showing their ability to self-renew. Bar scale represents 50 μm. Data represents the mean ± S.D. (**D**) Immunofluorescence staining shows a significant Ki-67, K14, ARF6 and RoR expression in the spheres formed by S2B11 primary tumor cells and in the tumor tissue itself. (**E**, **F**) Mammosphere formation ability (Bar scale represents 50 μm) (E) and migration capacity (Bar scale represents 25 μm) (F) was evaluated in S2B11 primary tumor cells after knocking-down the RoR with shRoR. Data represents the mean ±S.D (*n* = 3); ^**^p < 0.01. (**G**, **H**, **I**) Invasion capacity (Bar scale represents 25 μm) (G), migration capacity (Bar scale represents 25 μm) (H) and mammosphere formation ability (Bar scale represents 50 μm) (I) of S2B11 primary tumor cells was evaluated after knocking-down the miR-10b with sponge miR-10b. Data represents the mean ±S.D (*n* = 2); ^*^p < 0.05, ^**^p < 0.01

Finally, we wanted to confirm our *in vitro* findings *in vivo*. S2B11 cells (1 × 10^5^, 1.5 × 10^5^ or 2 × 10^5^) and S2D10 cells (1.5 × 10^5^ or 2 × 10^5^) were injected into the mammary glands of athymic nude mice, and tumor formation was monitored. All mice injected with S2B11 cells formed tumors including the ones injected with as few as 1 × 10^5^ cells, whereas no mice injected with S2D10 showed any tumor formation. Furthermore, the tumors of the mice injected with S2B11 cells had high levels of K14 expression, demonstrating the formation of basal tumors *in vivo* and the H&E staining showed that S2B11 cells formed more invasive tumors compared to the tumors formed by non-sorted DCIS cells (Figure 7A). We hypothesized that single-cell clone S2B11 was highly aggressive *in vitro* due to high self-renewal and maintenance of the CD49f^+^/CD44^+^/CD24^−^ signature. To confirm that tumors formed from S2B11 cells contained a high proportion of stem-like cells, we isolated primary cells from S2B11 tumors and performed FACS analysis. The results showed that CD49f^+^/CD44^+^/CD24^−^ cells were 13.6% of the whole tumor cell population (Figure 7B). We next tested the self-renewal of S2B11 tumor primary cells using mammosphere formation and serial re-plating. Primary S2B11 tumor cells were able to form high numbers of second and third generation mammospheres, demonstrating high levels of stem-like cells within this population (Figure 7C). Finally, using immunofluorescence, we found that both spheres formed from primary S2B11 tumor cells and the tumor tissue itself expressed high levels of Ki-67, K14, ARF6 and RoR (Figure 7D). Next, we wanted to confirm our findings linking RoR and miR-10b to the enhanced self-renewal, migration and invasion of single-cell derived clones in S2B11 primary tumor cells. We knocked-down RoR and miR-10b, and measured the self-renewal, migration, and invasion abilities of S2B11 primary tumor cells. When either RoR or miR-10b was knocked down in primary tumor cells, their migration (Figure 7E and Figure 7G) and mammosphere formation (Figure 7F and Figure 7I) were significantly decreased. Furthermore, knockdown of miR-10b significantly decreased the invasive capacity of the tumor cells (Figure 7H).

## DISCUSSION

Whole cell population-based research identifies average molecular expression and functional abilities throughout a differential population, and fails to discriminate subsets of cancer cells that are responsible for specific behaviors. Two recent studies used single-cell approaches to identify the tumorigenic and drug resistant single cell derived clones in GBM tumors and detect the early stage metastatic cells showing a distinct stem cell-like gene expression [8–9]. To our knowledge our study is the first to use a single-cell approach to study and characterize breast cancer subpopulations generated from clonal expansion of single cells sorted according to CD49f^+^/CD44^+^/CD24^−^ breast cancer stem-like cell markers. Through the clonogenic expansion of MCF10DCIS single-cells we identified and characterized the tumorigenic clones derived from CD49f^+^/CD44^+^/CD24^−^ single-cell sorting. Our functional studies showed that although we used 3 markers to sort them, the single cell derived clones still differed from each other significantly in a wide array of characteristics confirming the heterogeneity of the basal-like DCIS cells. Data presented here are essential for interrogating the molecular mechanisms underlying DCIS progression and identifying novel targets for developing new therapies.

Our data revealed that the more aggressive clones within DCIS cells have lower global DNA methylation compared to less aggressive clones and have enhanced expression of stem-cell, proliferation and invasion related genes and non-coding RNAs including SOX2, OCT4, K14, ARF6, Ki67, RoR and miR-10b. These results suggest that global DNA hypomethylation and specific activation of certain genes might be one of the early steps required for tumor initiation and progression in DCIS. Identification of the specific genes that are methylated in cancer progression will be an important next step for developing specific and efficient epigenetic therapies, as the response to anticancer drug likely differs depending on the methylation status of specific genes.

We also showed that activation of SOX2/OCT4 in the aggressive clones compared to non-aggressive ones using a dual reporter system. It is likely that aggressive clones are using the SOX2/OCT4 signaling pathway for self-renewal and maintenance. We also found that overexpression of lincRNA-RoR increases the stemness and migration of aggressive clones. This finding is not surprising considering the role of lincRNA-RoR in the regulation of pluripotency, and its association with the stemness transcription factors SOX2 and OCT4 [19–21]. Our data confirm the previous findings showing the association of lincRNA-RoR with SOX2 and OCT4 and suggest that activation of SOX2/OCT4/lincRNA-RoR signaling axis in specific subpopulations of DCIS cells might render them more aggressive.

We performed a PCR array for 84 miRNAs known or predicted to be associated with breast cancer. We found that five miRNAs were significantly upregulated (miR-429, miR-200a, miR-200b, miR-200c and miR-10b) and one of them was significantly downregulated (miR-29b) in aggressive clone compared to non-aggressive one. MiR-29b was shown to have higher expression in luminal breast cancer models compared to basal models and lower expression of miR-29b was associated with increased metastatic ability [25]. Breast cancer has a multi-step progression from epithelial accumulation to DCIS and finally transition to IDC [23] and same genes and miRNAs might show a different expression pattern depending on the stage of the disease. MiR-200 family is an example to such miRNAs. MiR-200 family regulates epithelial-to-mesenchymal transition (EMT) and mesenchymal-to-epithelial transition (MET) through the inhibition of ZEB1 and ZEB2 [26]. MiR-200b, miR-200c and miR-429 were shown to be upregulated in DCIS lesions compared to normal breast tissues [23]. We have observed that four members of miR-200 family were upregulated in our aggressive clones. This upregulation may be a key event in the aggressive clones of heterogeneous DCIS lesions, and the specific roles of the miR-200 family in our aggressive clones and their contribution to disease progression will be the focus in our future studies. Consistent with earlier reports, our data confirmed that miR-10b increases the mammosphere formation, migration and invasion ability of the aggressive clones [23, 24].

In summary, we show that the aggressive clones derived from CD49f^+^/CD44^+^/CD24^−^ single cells activate the OCT4/SOX2/lincRNA-RoR signaling axis to maintain cancer stem cell self-renewal and regulate differentiation. Finally, enhanced expression of K14, ARF6, and miR-10b helps these specific clones for migration and invasion. Identification and characterization of aggressive clones within DCIS stem cell population may benefit for the development of potential therapeutics to inhibit DCIS and DCIS-IDC transition.

## MATERIALS AND METHODS

### Cell culture and reagents

MCF10DCIS (DCIS) cells (Asterand) were cultured in DMEM/F12 supplemented with 5% heat-inactivated horse serum (Invitrogen), 4 μg/ml insulin (Gibco), 100 ng/ml cholera toxin, 0.5 μg/ml hydrocortisone (Sigma), and 20 ng/ml EGF (Life Technologies). Cells were incubated in 5% CO2 at 37°C.

### Flow cytometry and single cell sorting

After MCF10DCIS cells reached ~80% confluence, they were trypsinized, filtered with a 40 μm cell strainer (Fisher Scientific), and incubated with CD44/FITC (BD Biosciences, Cat No. 555478), CD49f/APC (eBioscience, Cat No. 17-0495-80) or CD24/PE (BD Biosciences, Cat No. 555428) antibodies and 0.5 μg/ml propidium iodide (Sigma) at 4°C for 30 min. CD44^+^/CD49f^+^/CD24^−^ cells that did not take up PI were then sorted into 96-well plates at 1 cell/well using 70 μm nozzle and incubated overnight at 37°C. On day one post-sorting, the plate was monitored under a bright-field microscope equipped with an X-Y stage to confirm that each well contained only one cell. The wells that contained more than one cell or no cells were excluded and the wells containing single cells were monitored everyday.

### Mammosphere assays

Single cells were obtained using 40 μm cell strainers (Fisher Scientific) and for mammosphere formation 1000–3000 ells/ml were seeded in six-well plates coated with 2% polyhema (Sigma) in DMEM/F12 containing 2% B27, 20 ng/ml EGF, 4 μg/ml insulin, and 0.4% BSA. After 7 days of culture, spheres larger than 100 μm were quantified by light microscopy. 15 fields/well were counted for each condition and average of 3 independent experiments were used to apply statistical analyses.

### Transwell invasion and migration assays

Transwell invasion and migration assays were performed using transwell migration chambers with 8-μm pore size (Costar, Cat No. 3422). For invasion, the upper chamber was coated with matrigel (Corning, Cat No. 354234) that was diluted to 3 mg/ml and incubated for 1 hr at 37°C. 1–2 × 10^5^ cells were then seeded in 200 μl serum-free medium to the upper chamber. The receiver contained 600 μl of complete growth medium with 10% horse serum. After 24 h the upper chambers were stained with 1% crystal violet solution and the invaded cells were quantified using light microscopy. For migration, 1.5–2.5 × 10^4^ cells were seeded in 200 μl serum-free medium to the uncoated upper chamber of the transwell. The receiver contained 600 μl of complete growth medium with 10% horse serum. After 16 h the upper chambers were stained with 1% crystal violet solution and the migrated cells were quantified using light microscopy.

### Detection of global DNA methylation

Colorimetric Methylated DNA Quantification Kit (Abcam, Cat No. 117128) was used to detect the global DNA methylation of the cells and the assay was performed according to manufacturer's recommendation.

### Detection of ALDH activity

ALDH activity of the samples was detected using the Aldehyde Dehydrogenase Activity Colorimetric Assay Kit (Sigma, Cat No. MAK082) following manufacturer's protocol.

### Cell cycle analysis

Cells were harvested and washed twice with 1X PBS before they were fixed in 70% ethanol at 4°C. At the day of analysis, the cells were centrifuged and washed once with 1X PBS. Flow cytometry was performed immediately after the cells were incubated with PI solution for 30 min at RT.

### 3D invasion assay

Wells of 96-well plates were coated with 0.75% agarose and cooled to room temperature. 5000 cells/well were plated in total volume of 100 μl culture medium. The cells were allowed to aggregate overnight by incubating in 5% CO_2_ 37°C. Next day after confirming the sphere formation, ECM solution was prepared by mixing matrigel (final concentration of 4.5–6 mg/ml) with culture medium. 100 μl of ECM solution was pipetted to a pre-chilled 96 well plate. One spheroid was embedded gently into one well of ECM. The plate was then placed into the incubator for 30 min. After 30 min incubation 100 μl of pre-warmed medium was added to each well. The plate was returned to incubator and the invasion was monitored by microscopy at desired intervals.

### Western blotting

Total cell lysates (20 μg) were separated by SDS-PAGE and blotted onto polyvinylidene difluoride membrane. The membrane was incubated with specific primary antibody overnight followed by the horseradish peroxidase (HRP)-conjugated secondary antibody, and visualized by the ECL Western blotting detection system (Thermo Scientific). β-actin (Sigma, Cat No. A5441) was used as the loading control. Antibodies against SOX2 (Cat No. ab97959) and OCT4 (Cat No. ab19857) were purchased from Abcam. Antibodies against SOX9 (Cat No. ab5535) and PARP1 (Cat No. sc-7150) were purchased from Millipore and Santa Cruz, respectively.

### Immunofluorescence staining and immunohistochemistry

Cells fixed in 8-well chamber slides with 4% ice-cold paraformaldehyde and formalin fixed and paraffin-embedded sections were used for immunofluorescence staining as previously described [5]. Samples were incubated with primary antibodies overnight followed by fluorochrome–conjugated secondary antibodies (Life Technologies) and DAPI counterstaining. Polyclonal rabbit anti-Ki67 (Cat No. sc-15402) and monoclonal mouse anti-ARF6 (Cat No. sc-7971) was purchased from Santa Cruz and polyclonal rabbit anti-K14 antibody was purchased from Covance (Cat No. PRB155P). Formalin fixed and paraffin-embedded sections were prepared for immunohistochemistry staining as previously described [27]. K-14 primary antibody was applied and followed by a biotin conjugated donkey anti-goat or goat anti-rabbit secondary antibody (Santa Cruz). Avidin-biotin peroxidase substrate kit (Vector Laboratories) was used to develop brown precipitate. Hematoxylin was utilized for nuclei staining.

### Fluorescence *in situ* hybridization of lincRNA-RoR

Fluorescence *in situ* hybridization of lincRNA-RoR was performed as previously described [16]. Cy3-labed lincRNA-RoR probe was obtained from Exiqon. Tissue sections were fixed in 4% formaldehyde and permeabilized using 0.5% Triton-X-100 in PBS, followed by blocking with 3% BSA in 4X saline–sodium citrate buffer. Tissue sections were hybridized overnight at 56°C with lincRNA-RoR probes (2 ng/mL dilution in buffer containing 10% dextran sulfate in 4X saline–sodium citrate buffer).

### Quantitative real-time PCR (qRT-PCR), transfection and Real-time PCR-based miRNA expression profiling

For qRT-PCR, total RNA was extracted with TRIzol reagent (Invitrogen) and analysis of mRNA/lncRNA expression was performed as described previously with normalization to either GAPDH or b-actin for mRNAs and to U6 small nuclear RNA for miRNAs [27]. Cells were transfected with SOX2/OCT4 reporter vector [18] with Lipofectamine 3000 (Invitrogen) according to the manufacturer's instructions. HD Fugene (Promega) was used to transfect the cells with shRoR, sponge-miR-10b and RoR and miR-10b overexpressing plasmids. shRNA for lincRNA-RoR was purchased from Origene. pBabe–lincRNA-RoR (plasmid 45763) [19], pBabe-puro-miR-10b sponge (plasmid 25043) [28] and MDH1-PGK-GFP miR-10b (plasmid 16070) [24] were purchased from Addgene. 96-well Human Breast Cancer miRNA PCR Arrays (Qiagen, Cat No. MIHS-109ZF-12) was performed to analyze the differential expression of 84 miRNAs known or predicted to be associated with breast cancer, according to the manufacturer's instructions.

### Xenograft studies

Single cell derived DCIS clones were collected, washed twice with cold PBS, mixed with matrigel 1:1 ratio and injected into mammary gland of 6 weeks old immunodeficient Nu/Nu female mice (University of Maryland School of Medicine, Veterinary Resources). Tumor growth was monitored weekly by caliper measurements (tumor size = (*L* × *W*^2^) × 0.5)), where *L* is the length and *W* is the width of each tumor. Studies were conducted under animal protocols approved by the University of Maryland School of Medicine/Animal Care and Usage Committee (ACUC).

### Statistical analysis

Statistical analysis was performed using the Graph Pad Prism software and data were assessed either by the 2-tailed Student t test or one-way ANOVA test for the correlation analyses. A difference was considered significant when *P* < 0.05 (*) or *P* < 0.01 (**). Data are presented as mean ± S.D.
